# 53BP1 Accumulation in Circulating Tumor Cells Identifies Chemotherapy-Responsive Metastatic Breast Cancer Patients

**DOI:** 10.3390/cancers12040930

**Published:** 2020-04-09

**Authors:** Fabienne Schochter, Kim Werner, Cäcilia Köstler, Anke Faul, Marie Tzschaschel, Barbara Alberter, Volkmar Müller, Hans Neubauer, Tanja Fehm, Thomas W.P. Friedl, Bernhard Polzer, Wolfgang Janni, Brigitte Rack, Lisa Wiesmüller

**Affiliations:** 1 Department of Obstetrics and Gynecology, Ulm University, 89075 Ulm, Germany; fabienne.schochter@uniklinik-ulm.de (F.S.); kim.werner@uniklinik-ulm.de (K.W.); anke.faul@uniklinik-ulm.de (A.F.); marie.tzschaschel@uniklinik-ulm.de (M.T.); thomas.friedl@uniklinik-ulm.de (T.W.P.F.); wolfgang.janni@uniklinik-ulm.de (W.J.); brigitte.rack@uniklinik-ulm.de (B.R.); 2 Division of Personalized Tumor Therapy, Fraunhofer-Institute for Toxicology and Experimental Medicine, 93053 Regensburg, Germany; caecilia.koestler@item.fraunhofer.de (C.K.); Barbara.Alberter@item.fraunhofer.de (B.A.); bernhard.michael.polzer@item.fraunhofer.de (B.P.); 3 Department of Gynecology, University Medical Center Hamburg-Eppendorf, 20246 Hamburg, Germany; v.mueller@uke.de; 4 Department of Obstetrics and Gynecology, University of Duesseldorf, 40225 Duesseldorf, Germany; Hans.Neubauer@med.uni-duesseldorf.de (H.N.); Tanja.Fehm@med.uni-duesseldorf.de (T.F.)

**Keywords:** metastatic breast cancer, circulating tumor cells, triple-negative, hormone receptor, Eribulin, predictive biomarker, progression-free survival

## Abstract

Evidence suggests that the DNA end-binding protein p53-binding protein 1 (53BP1) is down-regulated in subsets of breast cancer. Circulating tumor cells (CTCs) provide accessible “biopsy material” to track cell traits and functions and their alterations during treatment. Here, we prospectively monitored the 53BP1 status in CTCs from 67 metastatic breast cancer (MBC) patients with HER2- CTCs and known hormone receptor (HR) status of the primary tumor and/or metastases before, during, and at the end of chemotherapeutic treatment with Eribulin. Nuclear 53BP1 staining and genomic integrity were evaluated by immunocytochemical and whole-genome-amplification-based polymerase chain reaction (PCR) analysis, respectively. Comparative analysis of CTCs from patients with triple-negative and HR+ tumors revealed elevated 53BP1 levels in CTCs from patients with HR+ metastases, particularly following chemotherapeutic treatment. Differences in nuclear 53BP1 signals did not correlate with genomic integrity in CTCs at baseline or with nuclear γH2AX signals in MBC cell lines, indicating that 53BP1 detected features beyond DNA damage. Kaplan–Meier analysis revealed an increasing association between nuclear 53BP1-positivity and progression-free survival (PFS) during chemotherapy until the final visit. Our data suggest that 53BP1 detection in CTCs could be a useful marker to capture dynamic changes of chemotherapeutic responsiveness in triple-negative and HR+ MBC.

## 1. Introduction

Triple-negative breast cancer (TNBC), which is diagnosed in 10–15% of all MBCs, affects younger patients and patients with a mutation in BRCA genes more frequently. With a median survival of 13.3 months after diagnosis of metastatic sites, TNBC has a particularly poor prognosis. Often patients develop a resistance to first-line chemotherapy very rapidly, resulting in a median duration of first-line palliative chemotherapy of 11.9 weeks [1].

Triple-negative tumors are characterized by the lack of HR and HER2 expression, but aside from this common feature this subgroup includes very heterogeneous tumors. A mutation in *BRCA1* or *2* is found in up to 20% of triple-negative metastatic patients [2]. The proteins encoded by BRCA genes are critically involved in DNA double-strand break (DSB) repair, more specifically, in the error-free pathway of homologous recombination repair (HRR) [3]. In TNBC, a high prevalence of gene mutations as well as epigenetic changes result in BRCAness, compromising safe DNA repair through HRR [2,4,5]. The DNA damage response factor 53BP1 is crucial in protecting DNA ends in BRCA1-defective cells from resection and entry into error-prone repair pathways [6,7,8]. It has been demonstrated that 53BP1 expression in breast cancer is associated with poor prognosis, particularly in TNBC frequently showing BRCA1 dysfunction [7,9].

Due to the lack of predictive targets, chemotherapy was the only treatment option available for a long time. This results in an urgent clinical need for new therapies. During the last years, new drugs such as the Poly(ADP-ribose) polymerase (PARP)-inhibitors targeting HRR-defective tumors were studied in several clinical trials. Two different phase III trials (OlympiaAD and EMBRACA) showed an improved response rate and PFS for PARP inhibitor (Olaparib or Talazoparib)-treated patients compared to patients who received standard chemotherapy [10,11]. Among the new therapeutics Eribulin, a non-taxane microtubule inhibitor, demonstrated an improved overall survival (OS) in patients with MBC already treated with taxane and anthracycline compared to treatment with physicians’ choice in the EMBRACE trial [12]. A pooled analysis by Pivot and colleagues [13] further revealed the benefit for the triple-negative subgroup of patients. Shimomura et al. [14] suggested *BRCA* mutation as a potential biomarker for the combination of Eribulin and Olaparib.

With new therapeutic options, there is an even more urgent need for new biomarkers, serving to improve personalized and target-directed therapy in this heterogenous group of patients. Circulating tumor cells already proved their prognostic relevance in the adjuvant setting and MBC [15,16,17]. While CTC dynamics during treatment predicts the therapy response [18], so far no clinical trial using the number or the dynamics of CTCs as a predictive value provided evidence for a clinical benefit [19]. It seems even more interesting to use CTCs to define subgroups [20] and use their biological information as a surrogate for therapeutic response [21]. In this study, we monitored 53BP1 as a parameter for an intact DNA damage response in CTCs from both metastatic triple-negative and HR+ breast cancer patients and determined its predictive value.

## 2. Results

### 2.1. Detection of 53BP1 Signals in CTCs from MBC Patients

**Figure 1:**
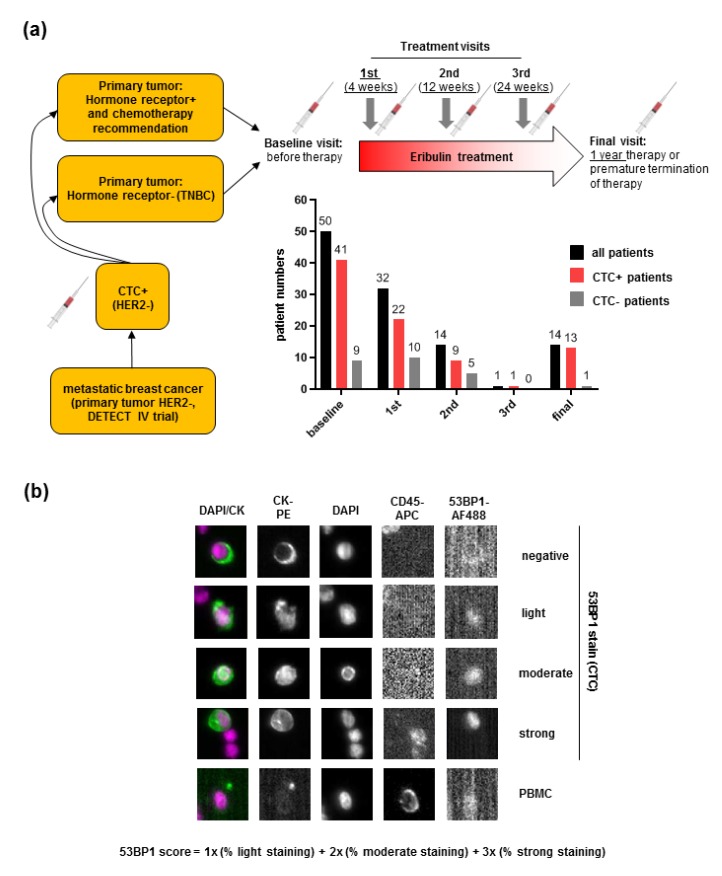
Detection of 53BP1 in CTCs from MBC patients in the DETECT IV trial. (**a**) Selection of MBC patients with CTC-positivity (HER2-) and primary TNBC or primary HER2-, HR+ breast cancer for Eribulin monotherapy and blood sample collection. Blood samples were drawn before therapy (baseline visit), four, twelve, and 24 weeks after the start of Eribulin treatment (1st, 2nd, and 3rd treatment visit) as well as one year after treatment initiation or during premature termination due to disease progression (final visit). The numbers of patients recruited at the different visits are indicated by black columns, the numbers of CTC+ and CTC- patients among them by red and grey columns, respectively. Note that not all of the patients described in Table 1 entered the study during the baseline visit but during later visits which explains the differences in patient numbers. (**b**) Evaluation of 53BP1 signals in CTCs. Following EpCAM-based CTC enrichment and immunofluorescence microscopic imaging (Cellsearch^®^), CTCs (CK+, CD45-) were enumerated and 53BP1-staining intensity assessed per individual CTC as indicated by representative examples. A 53BP1 score per patient sample was calculated from the percentage of CTCs without and with light, moderate or strong staining. PBMC, peripheral blood mononuclear cell (CK-, CD45+).

Accumulating evidence has demonstrated that loss of 53BP1 expression in breast cancer is associated with poor prognosis, particularly when focusing on TNBC patients [7,9]. Therefore we aimed at detecting 53BP1 in CTCs of MBC patients with defined HER2 and HR status to explore its potential as a biomarker. To this end, we collected blood samples from CTC-positive (CTC+) MBC patients with HER2-negative (HER2-) primary tumors and included patients with HER2- CTCs in a translational project in the course of the DETECT IV trial [22] (Figure 1a). CTC enrichment, enumeration, and image analysis were performed by the EpCAM-based Cellsearch^®^ technology [15]. CTC-positivity (≥1/7.5 ml blood) as well as the HER2 status were determined based on established morphological and immunocytochemical criteria following nuclear (DAPI), cytokeratin (CK), CD45, and HER2 immunostaining [23]. For comparative analysis of CTCs from TNBC and non-TNBC patients, we recruited a total of 67 MBC patients with known HR status of the primary tumor and/or metastases. Based on the HR status determined for the primary tumor (N = 63), 48 and 15 patients had HR+ and triple-negative tumors, respectively. HR status of metastases was known for 43 patients, with 29 HR+ and 14 triple-negative tumors (see Table 1 for patient characteristics). Eribulin monotherapy was administered to all patients. For our study, blood samples were drawn during the baseline visit before therapy intiation and during the 1st, 2nd, and 3rd visits four, twelve, and 24 weeks after treatment initiation, respectively. Additional blood was sampled during the final visit at the regular end of treatment after one year or due to premature termination. 

As expected, we found a decline of mean CTC numbers from baseline to twelve weeks of treatment but a dramatic rise at the final visit due to disease progression in 10/13 of the cases (mean values at baseline: 18, 2nd visit: 2, final visit: 118) (Supplementary Figure S1). Detection of nuclear 53BP1 was achieved by transfer of the staining technology established for immunofluorescence microscopy of patient-derived cell lines [24] to the Cellsearch^®^ system. To semiquantitatively evaluate 53BP1 levels per patient sample, we calculated a 53BP1 score according to the formula in Figure 1b. In an earlier study, such a score provided a reliable measure for immunohistochemical 53BP1 staining in breast cancer tissue arrays [25]. In our study, the 53BP1 score enabled us to integrate differential immunoreactivities for all CK+, CD45- CTCs from each sample. Longitudinal analysis of the mean 53BP1 scores for CTCs from MBC patients (Figure 1a) did not reveal statistically significant differences before and after Eribulin monotherapy (Supplementary Figure S1), suggesting that the drug did not unleash a significant 53BP1 response in general. 

### 2.2. 53 BP1 Accumulates in CTCs from HR+ MBC Patients During Eribulin Treatment

**Figure 2:**
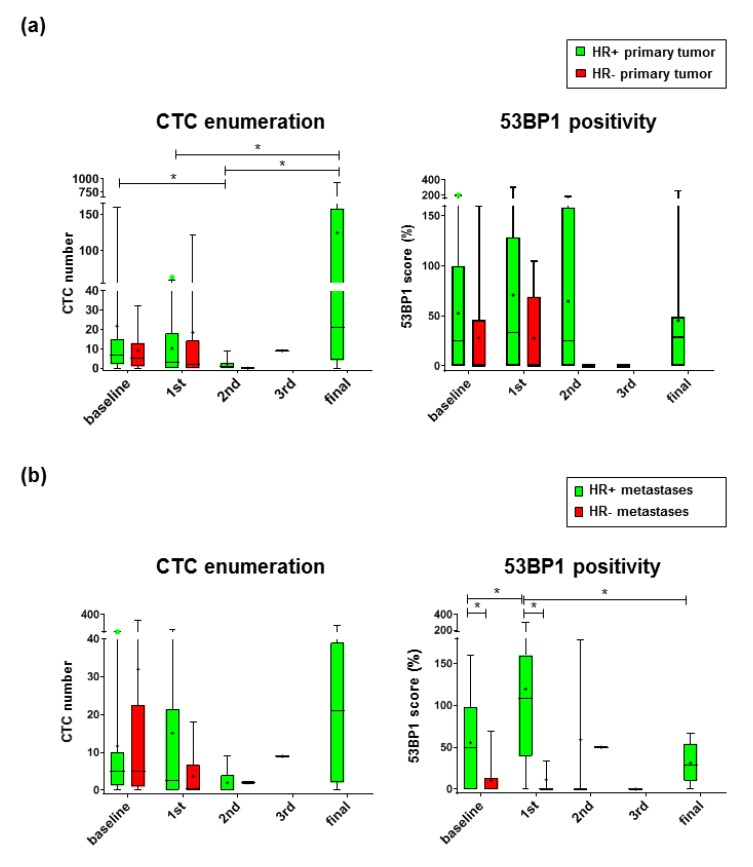
Comparison of CTCs as a function of the HR status in primary tumors or metastases. CTCs from patients with HR+ and HR- primary tumors (**a**) or metastases (**b**) were enumerated and 53BP1 scores determined. Data are shown in box plots with the mean value (dot), median (line), and 95% confidence intervals (CI) (whiskers). For numbers of independent blood samples (N) obtained during different visits, see Supplementary Figure S2. * *P* < 0.05, Kruskal–Wallis test followed by the Mann–Whitney U test. For individual patient scores, see Supplementary Figure S2.

Given that a higher prognostic value of the 53BP1 status in tumor tissue had been reported for TNBC patients [26], we compared CTCs from HR-, i.e., TNBC, and HR+ MBC patients who otherwise featured comparable clinical characteristics (Table 1). Among the MBC patients recruited during the baseline and the 1st treatment visits, more than one fourth had suffered from an HR- primary tumor (Supplementary Figure S2). None of these TNBC patients participated in the trial until the final visit, which was mostly due to adverse events or disease progression, illustrating the poor prognosis of this breast cancer subtype [27]. Similar to the pattern seen with all MBC patients (Supplementary Figure S1), mean CTC numbers in MBC patients with primary HR+ breast cancer declined from the baseline to the 2nd treatment visit and then increased again until the final visit (Figure 2a). In MBC patients with primary TNBC, differences were not seen between mean CTC numbers or 53BP1 scores at different visits. There were also no statistically significant differences found between the values for MBC patients with primary HR+ and HR- breast cancer. 

When we focused on MBC patients with different HR status of the metastatic lesions (Supplementary Figure S2), we noticed higher 53BP1 scores in CTCs from MBC patients with HR+ as compared with HR- metastases at the baseline and 1st treatment visits (5.2- and 10.8-fold, respectively; Figure 2b). Interestingly, on average, 53BP1 signals increased in CTCs from MBC patients with HR+ metastases from the baseline to the 1st treatment visit (2.2-fold) and then returned to below the baseline level until the final visit (3.8-fold), while 53BP1 scores stayed low in the case of HR- metastases. In comparison, CTC numbers were not statistically significantly different between the MBC patient groups or between visits. In conclusion, 53BP1 staining of CTCs from MBC patients with HR+ metastases showed dynamic changes during Eribulin monotherapy, but was close to the detection limit in the case of HR- metastases, both at the baseline and in treatment visits.

### 2.3. Reduced Formation of Nuclear 53BP1 Foci in TNBC Cell Lines Does Not Reflect Changes in γH2AX-Labeled DNA Damage

**Figure 3:**
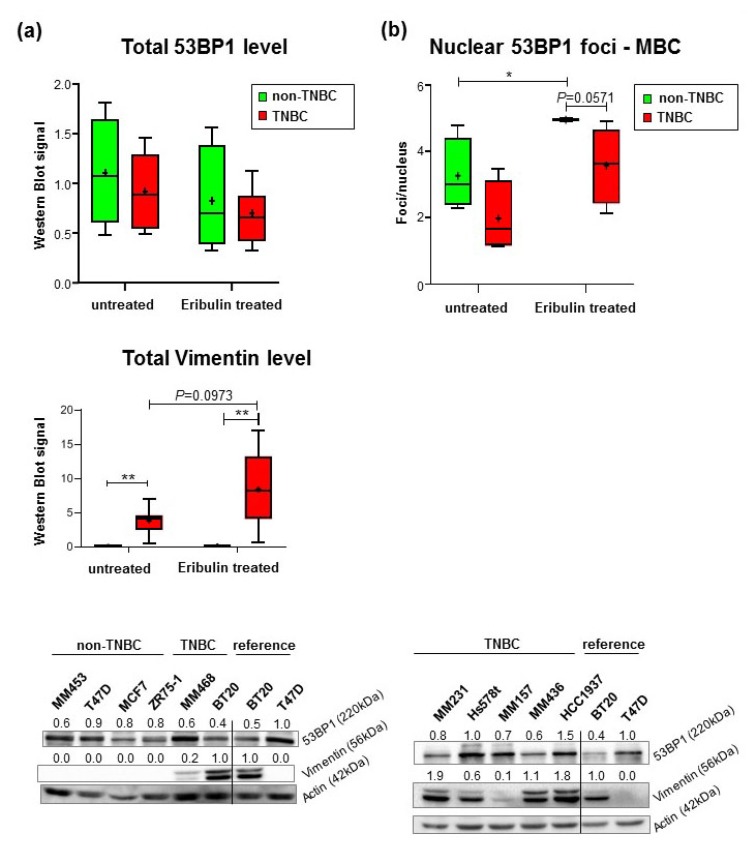
Protein expression and nuclear foci formation in breast cancer cell lines. (**a**) Cellular protein levels. 53BP1 and Vimentin band intensities were normalized to loading controls. Values for T47D cell extracts, loaded as a reference on each blot, were set to 1. Mean relative Western blot signals per cell line were calculated from two independent experiments. Box plots in the upper panels show mean values (cross), the median (line), and 95% CI (whiskers) for untreated and for 7d Eribulin-treated non-TNBC (N=4) and TNBC cell lines (N=7). The Kruskal–Wallis test did not reveal significant differences in the case of 53BP1. Representative immunoblots are shown in the bottom panels. Vimentin staining was detected in all TNBC but was negative in all non-TNBC cell lines in which BT20 served as a reference. Actin normalized protein levels are indicated above the 53BP1 and Vimentin signals. (**b**) Focal accumulation of 53BP1 in the nucleus of MBC cell lines. 53BP1 foci were detected by immunofluorescence microscopy in MBC cell lines. The graph presents quantitative data in box plots as in A; ~300–900 nuclei per sample were scored in two independent experiments each, and mean values were calculated for each cell line. *P<0.05, Kruskal–Wallis test, Mann–Whitney U test. Representative immunofluorescence images are displayed in Supplementary Figure S3.

To better understand differential 53BP1 staining observed in CTCs, we analyzed 53BP1 in a panel of seven TNBC and four non-TNBC cell lines. As expected [28], all TNBC cell lines expressed Vimentin, a marker for epithelial mesenchymal transition (EMT), whereas all non-TNBC cell lines were devoid of Vimentin (Figure 3a). Mean Vimentin Western blot signals in Eribulin-treated TNBC cell lines showed a trend of increased levels (*P* = 0.0973). Then, we assessed total 53BP1 levels in cellular extracts by Western blotting, which did not reveal statistically significant differences between the mean values for these two groups or for cells treated with or without Eribulin for 7 d. Next, we engaged immunofluorescence microscopy to analyze the cellular localization of 53BP1. In this way, we observed characteristic focal 53BP1 signals in the nuclei of both cell types (Supplementary Figure S3). Quantification of nuclear 53BP1 foci demonstrated elevated numbers in non-TNBC versus TNBC cells before Eribulin treatment (1.8-fold) and Eribulin-induced foci accumulation in both breast cancer cell types (1.5- to 2.0-fold) (Supplementary Figure S3). Notably, measurements of IC50 values by MTT following Eribulin treatment did not reveal statistically significant differences between the two groups of cell lines (IC50 = 1 μM, data not shown). Cell lines from MBC only (non-TNBC cell lines: MM453, T47D, MCF7, and ZR75-1; TNBC lines: MM468, MM231, MM157, and MM436) confirmed the Eribulin treatment-induced rise; however, non-TNBC cells only revealed a trend of higher foci numbers in non-TNBC versus TNBC cells after treatment (1.4-fold, *P* = 0.0571) (Figure 3b). 53BP1 binds DSBs and protects them from the DNA end resection machinery [6,7]. To understand whether the observed changes in 53BP1 foci numbers reflect the accumulation and/or removal of DNA lesions such as DSBs, we re-analyzed the panel of breast cancer cell lines for the appearance of the DNA damage marker γH2AX. Surprisingly, neither in mock- nor in Eribulin-treated cells were γH2AX foci numbers found to differ between TNBC and non-TNBC cell lines (Supplementary Figure S3), also when focusing on MBC cell lines (data not shown). These results suggested that the decline of nuclear 53BP1 signals seen in metastatic TNBC cells did not simply reflect a general decrease in chromosomal DNA damage and therefore may indicate a feature associated with TNBC independent of DSB levels. 

### 2.4. 53 BP1 Signals Rise in CTCs from MBC Patients with Low Genomic Integrity During Eribulin Treatment

**Figure 4:**
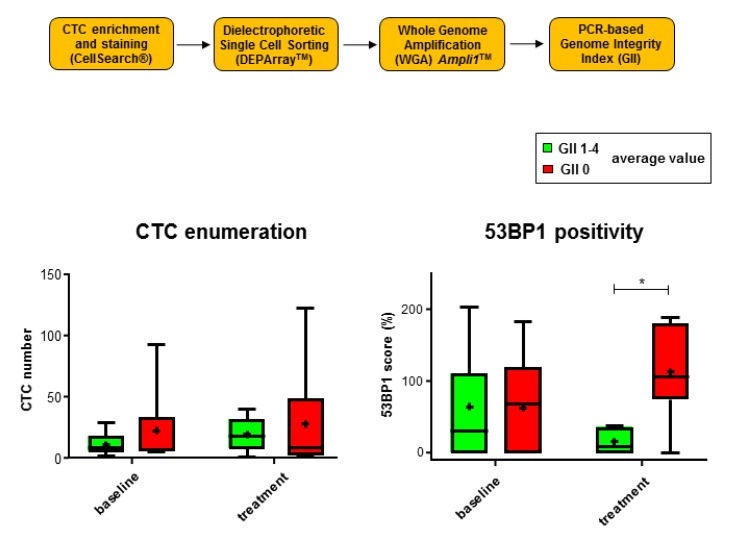
Analysis of genomic integrity as a function of 53BP1 scores of CTCs. The workflow for the determination of the genomic integrity index (GII) following CTC enrichment, staining, single cell sorting, whole genome amplification (WGA), and PCR is schematically drawn on the top. GII values were determined for single CTCs, and average values per sample were calculated. The lower left graph displays CTC numbers with remaining versus loss of genomic integrity for blood samples collected during baseline (GII 1–4, N = 11, and GII 0, N = 9) and treatment visits 1-2 (GII 1–4, N = 5 and GII 0, N = 6). The lower right panel shows 53BP1 scores for these four groups. Box plots show mean values (cross), the median (line), and 95% CI (whiskers). * *P* < 0.05, Mann–Whitney U test.

For assessment of the chromosomal damage in CTCs from the MBC patients with differing 53BP1 staining intensity, we determined a genomic integrity index (GII) according to our previously established PCR-based protocol [25]. To this end, we isolated individual CTCs following image analysis by the Cellsearch^®^ system via dielectrophoretic single cell sorting using DEPArray™ technology (Figure 4). Individual CTCs were then subjected to whole genome amplification (WGA) and multiplex PCR for classification into five GII values; GII 0 indicated fully fragmented genomic DNA, GII 4 the absence of DNA damage. CTC enumeration did not reveal significant differences between patient samples with complete loss of genomic integrity in all CTCs (GII 0) as compared with DNA damage in at least a fraction of the CTCs (GII 1-4). We also did not observe differences between the 53BP1 scores of samples with complete loss of (GII 0) or remaining (GII 1-4) genomic integrity in CTCs at baseline. Conversely, after treatment (1st and 2nd treatment visits), we hardly detected 53BP1 signals in the CTCs from samples with remaining genomic integrity (GII 1-4), while 53BP1-positivity increased in CTC samples with GII 0 and therefore was 6.9-fold higher compared with GII 1-4 samples (Figure 4). When we discriminated between samples in which the highest values specific for single CTCs were GII 0-2 (PCR amplification of no more than one long fragment) versus GII 3-4 (at least two of the long PCR fragments amplified), we found a corresponding 6.9-fold difference post-treatment (Supplementary Figure S4). This comparison also revealed a treatment-induced 2.5-fold increase of 53BP1 scores in samples with low genomic integrity (GII 0-2), which was reminiscent of the 53BP1 signal rise in patients with HR+ metastases. 

Due to the small sample numbers when engaging this challenging technique of single cell sorting and WGA, a comparison following further stratification into HR+ and HR- groups was not meaningful. However, we recalculated the data for the larger subgroup, namely, for MBC patients with HR+ metastases (Supplementary Figure S4). The results for HR+ metastases showed a similar pattern as for all samples, suggesting that MBC patients with HR+ metastases were not simply identical to patients carrying CTCs with low or high GII. Importantly, we observed 53BP1 accumulation post-treatment only in patients with CTCs that showed overall low genomic integrity, highlighting Eribulin-dependent induction of 53BP1 signaling in tumor cells of this subgroup of MBC patients. 

### 2.5. 53 BP1 Score as a Biomarker for Progression-Free Survival of MBC Patients

**Figure 5:**
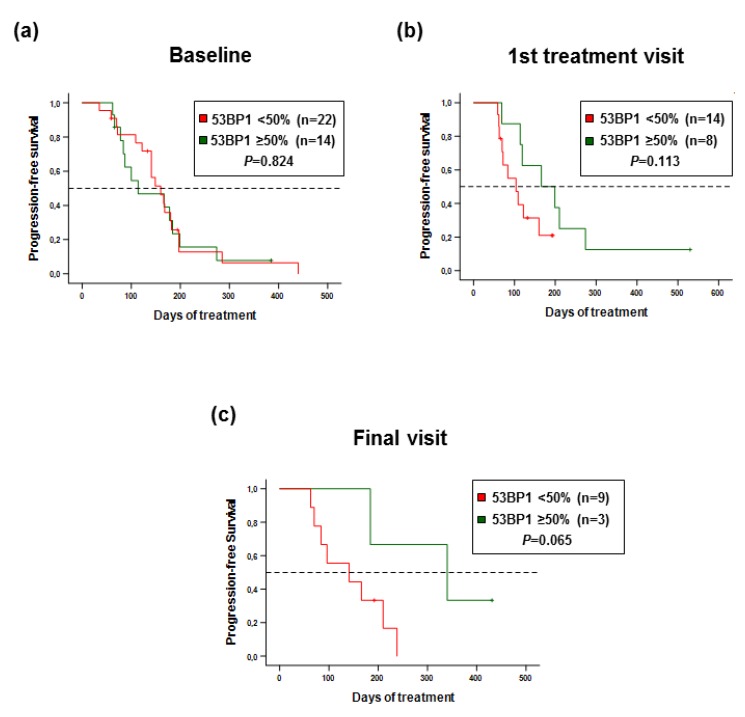
Longitudinal comparison of PFS curves based on 53BP1 status. Progression-free survival (PFS) curves are based on Kaplan-Meier estimates for MBC patients with CTCs showing a 53BP1 score <50% or ≥50%. (**a**) PFS of patients recruited during the baseline visit. (**b**) PFS of patients recruited during the 1st treatment visit. (**c**) PFS of patients recruited during the final visit.

To understand whether the observed 53BP1 responses to Eribulin in MBC patient subgroups, namely, in patients with HR+ metastases or with GII 0–2 CTCs, are indicative of treatment efficacy, we examined the power of 53BP1 scores obtained with blood-derived CTCs as a prognostic and/or predictive marker. We used mean 53BP1 scores in baseline CTC samples as dividing criteria, i.e. compared patients with 53BP1 scores <50% and ≥50%. We analyzed PFS of the patients from these two groups using scores obtained with samples at different time points during the study. The Kaplan–Meier curves in Figure 5 illustrate that 53BP1 scores obtained with samples from patients during the baseline visit did not reveal differences in PFS. However, patients with high 53BP1 scores obtained after the start of the treatment and during the final visits showed an increasing trend of longer PFS (1st treatment visits: *P* = 0.113; final visits: P = 0.065). Our data therefore support the concept that 53BP1 represents a surrogate marker for PFS of MBC patients undergoing chemotherapy. More specifically, our study highlights a potential application of 53BP1 detection on CTCs derived from blood samples to predict responsiveness to Eribulin monotherapy. 

## 3. Discussion

Circulating tumor cells have become the subject of intense research aiming at the detection of druggable features for personalized anticancer treatment approaches [29]. Previous studies revealed a prognostic value of 53BP1 detection in primary breast cancer [7,9,26]. Therefore, we considered whether in MBC patients, where CTCs are most prevalent, these cells are useful biopsies to monitor 53BP1 signals. Given that downregulation of 53BP1 expression was reported to associate with disease progression in a subgroup of TNBC patients [7], we compared CTC+ MBC patients with triple-negative and with HR+ primary tumor and/or metastases. Our two study subgroups within the DETECT study program were comparable in terms of mean patient age and BMI, histological type of the primary tumor, and the number of pre-treatments. Importantly, all patients in our study received the same chemotherapeutic treatment, namely, Eribulin monotherapy, providing maximally controlled conditions for a study of patients with otherwise extremely complex disease status due to the heterogeneous nature of their heavily pretreated malignancies.

Most known breast cancer predisposing gene mutations compromise accurate DSB repair by HRR [3,30]. HRR dysfunction has also been connected with the sporadic form of TNBC, as deleterious mutations and/or reduced expression of HRR genes were frequently observed [2,4,5]. 53BP1 is known to protect unrepaired DSBs from excessive resection in HRR dysfunctional cells, whereby alternative, mutagenic DSB repair mechanisms remain repressed [6,7,8]. This feature explains why reduced overall expression of 53BP1 in subsets of TNBC was found to be associated with poor prognosis and with resistance to PARP inhibitor therapies. Our results obtained with CTCs support this idea and suggest 53BP1 downregulation in TNBC with disease progression. Thus, while we did not find statistically significant differences between the 53BP1 scores for primary TNBC and HR+ breast cancer patients, we calculated lower values for HR- versus HR+ MBC. Notably, we immunodetected 53BP1 signals in the nuclear compartment of individual CTCs, where 53BP1 acts in DSB repair, while previous work relied on tissue array or mRNA expression array data [7,9]. When we compared total cellular 53BP1 expression and nuclear accumulation of 53BP1 in cell lines by Western blot and immunofluorescence microscopy, respectively, we noticed that the discriminatory power of changes in 53BP1 signals between cells from TNBC and HR+ tumors significantly increased with microscopic imaging. This result can be explained, e.g., by altered posttranslational regulation of the nuclear import of 53BP1, similarly as was previously shown for BRCA1 retention causing HRR dysfunction in sporadic breast cancer [31]. Therefore, we believe that microscopic imaging is superior to expression analysis for evaluation of the 53BP1 status in clinical samples.

Quite different from our observations, Nagelkerke and colleagues found an association between low γH2AX levels and disease-free survival of TNBC patients (not HR+ breast cancer patients) and a similar, but weaker trend for 53BP1 [26]. However, patients donating to the tissue microarray of this study were node negative and free from metastases and did not receive adjuvant systemic treatment but rather post-surgical radiotherapy. The authors interpreted their data such that the DSBs labeled by γH2AX in these primary tumors represent sites of failed repair attempts, causing persistence of the radiation-induced DSBs and promoting rearrangements. Adams et al. further noticed that another early DSB repair component, RAD50, can be detected on CTCs from primary lung cancer patients, but only once they undergo radiotherapy [32]. Importantly, our patients were MBC and not primary cancer patients treated by chemo- and not radiotherapy.

Nevertheless, the question may arise whether changes in 53BP1 signals observed here may simply have reflected altered DSB levels. Arguing against this possibility, we did not find the 53BP1-specific pattern of elevated nuclear foci numbers in Eribulin-treated HR+ MBC cell lines with the well-established DSB marker γH2AX [33]. Moreover, 53BP1 scores of CTCs correlated with a decline of genomic integrity, as determined by a PCR-based assay, only after the start of Eribulin treatment. At baseline, this readout for chromosome cleavage and fragmentation and thus DSBs did not discriminate between 53BP1-positive and negative CTCs. Therefore, 53BP1 positivity in CTCs unlikely reflected DSB numbers exclusively. From our results, we propose that downregulation of 53BP1 occurred in a fraction of patients during tumor progression towards triple-negative metastases and during chemotherapy-induced evolution of metastatic tumors to de-repress mutagenic repair for better tumor cell survival. Consequently, these patients with reduced 53BP1 showed reduced PFS.

Our study patients received Eribulin monotherapy independently of the HR status. Eribulin has multiple modes of action underlying its antiproliferative effect in cancer cells and survival benefit in clinical trials [34]. It is best known for its inhibitory effect on microtubule polymerization, thereby interfering with microtubule dynamics. Additionally, it has non-mitotic effects including tumor vasculature remodeling and reversal of EMT. TNBC cells frequently display EMT, i.e., acquisition of mesenchymal and loss of epithelial cell characteristics, which plays a crucial role in the release of CTCs with metastasizing potential and in the therapeutic response [28]. In our study, we engaged the well-established Cellsearch^®^ technology with EpCAM-based enrichment of CTCs [15]. We did not find significant differences between average numbers of CTCs from MBC patients with HR- and HR+ malignancies, even though we may have missed a fraction of CTCs potentially having undergone EMT-induced downregulation of EpCAM. However, the observed changes of 53BP1 signals on CTCs when comparing HR- versus HR+ MBC patients after treatment were confirmed in MBC cell lines providing a good estimate of the reliability of our results with CTCs. In this context, it is of interest that recent work demonstrated high plasticity between epithelial, EMT, and reverse MET phenotypes in breast cancer stem cells and suggested that cells with hybrid features are most proficient in reaching the circulation and forming metastases [35,36]. Therefore, EpCAM-based selection may capture a representative fraction of CTCs at least regarding their 53BP1 status, which, however, will need more systematic analysis in future trials on patient-derived CTCs.

Our data suggest that 53BP1 signal intensities in CTCs may represent a biomarker to monitor responsiveness during Eribulin treatment in MBC patients. Eribulin has not been demonstrated to directly induce DSBs which could be recognized by 53BP1. Of note, Poruchynsky et al [37] discovered that the microtubule-targeting agents vincristine and paclitaxel sequestered key DSB repair proteins including ATM, RAD50, and 53BP1 in the cytoplasm. However, we noticed an increase and not a decrease of nuclear 53BP1 in CTCs from Eribulin-treated HR+ MBC patients, which is why we consider Eribulin-induced cytoplasmic sequestration of 53BP1 unlikely. Intriguingly, however, Lottersberger and colleagues [38] demonstrated that 53BP1 requires dynamic microtubules connected to the nuclear envelope to promote the roaming of DSBs to increase the chances to reconnect. Therefore, we hypothesize that Eribulin-induced lack of mobility of DNA ends could be the reason for the apparent increase of 53BP1 signals in CTCs from HR+ MBC patients following treatment. In TNBC patients, 53BP1 was downregulated already before Eribulin treatment, namely, during progression towards metastasis.

Present treatments of MBC patients include conventional chemotherapeutics such as anthracyclines and taxanes, but hopefully in the near future, more effective and/or better tolerable drugs will be available [27,39]. Though not addressed in our present study, monitoring 53BP1 in CTCs may also identify resistance to PARP inhibitors as previously suggested from preclinical investigations [40]. Here, we demonstrate the feasibility of detecting nuclear 53BP1 in CTCs from MBC patients. It will therefore be interesting to evaluate the power of this marker to predict responsiveness of MBC patients to PARP inhibitor and other DNA repair-related drug treatments in future studies. Eribulin has become a promising drug for MBC, as it mediates improved OS in patients with pretreated MBC [41]. Predictive markers are needed for Eribulin treatment responses, but there are none available. Our work may lay a cornerstone for marker development to help in decision making for mono- and/or combined Eribulin chemotherapy recommendations for MBC patients.

## 4. Materials and Methods

### 4.1. Patient Recruitment and Sample Collection

The DETECT-program is a multicenter study with more than 100 sites in Germany. All 67 analyzed patients participated in the DETECT IV study arm B (ClinicalTrials.gov identifier NCT02035813; EudraCT-No. 2013-001269-18). Here, patients with locally advanced or metastatic and HER2 negative (HER2-) breast cancer are tested for CTCs.

The DETECT IV B study offers treatment with Eribulin to all patients with HER2- CTCs and indication to chemotherapy, i.e., patients with both HR+ or triple-negative tumors. All patients were more than 18 years of age and received up to three prior lines of therapy. Eribulin was administered in a single-arm non-randomized phase II observation. CTC follow-up assessment was performed after cycle 1 (3–4 weeks), cycle 3 (9–12 weeks), cycle 6 (21–24 weeks), and at the end of treatment.

All enrolled patients agreed with written informed consent. The study concept was in accordance with the Declaration of Helsinki and adhered to good clinical practice and German pharmaceutical law. The whole study concept was approved by the local ethics committees of the participating centers. The translational investigations were additionally approved by the DETECT-study leading ethics committee of the University of Düsseldorf (Study-no. MC-LKP-668).

### 4.2. CTC Enrichment, Immunostaining, and Image Analysis

For CTC identification, we used Cellsearch^®^ (Janssen Diagnostics, Raritan, NJ/Menarini Silicon Biosystems, Inc, Florence, Italy) technology following the standard operating procedures. Briefly, CTCs from two 7.5 ml CellSave tubes per visit were subjected to EpCAM-based ferrofluid selection, immunostaining, and image analysis, taking care to exclude apoptotic cells from CTC analysis in all samples [42,43]. One CellSave tube was used to identify patients with cytokeratin-positive (CK+), CD45-, and HER2- CTCs using phycoerythrin-conjugated antibodies recognizing epithelial cell-specific CKs (predominantly cytokeratins 8, 18, and 19), allophycocyanin (APC)-conjugated antibody directed against the white blood cell marker CD45, and 6-diamidino-2-phenylindole (DAPI) to stain nuclei. For HER2 immunodetection, we used the anti-Her2/neu antibody in the Cellsearch^®^ protocol (Menarini Silicon Biosystems) and determined HER2+ versus HER2- status as described before [44]. The second CellSave tube was engaged for the EpCAM-based selection and identification of CK+ and CD45- CTCs as before. However, the HER2-specific antibody was replaced by Alexa Fluor 488 (AF488)-conjugated anti-53BP1 antibody (NB100-304AF488, 1:50, Novus Biologicals, Centennial, CO, USA).

### 4.3. Evaluation of Genomic Integrity in Individual CTCs

Individual CTCs were isolated from Cellsearch^®^ cartridges after HER2 and 53BP1 image analysis using DEPArray™ technology exactly as described [45]. To assess the DNA integrity of single CTCs isolated from patient samples, we firstly amplified the genomic DNA with our workflow, using the *Ampli*1^TM^ QC Kit from Menarini Silicon Biosystems. Then, we used a PCR-based assay to evaluate the DNA integrity of samples after *Ampli*1^TM^ WGA Kit-based amplification. Four markers, designed for one short and three long DNA fragments, are multiplexed in one reaction, and the number of apparent bands is correlated to define a genome integrity index (GII). GII values range from 0 (no band detected) to 1 (only the short fragment detected), 2 (any one of the three long fragments detected), 3 (any two of the long fragments detected), and 4 (all three long fragments and/or not the small fragment detected) [45]. Cells with highest quality DNA typically produce three or four PCR bands, while cells with degraded DNA will show fewer bands. With this assay, we have a useful quality control check of the amplification procedure to assess the DNA integrity [45]. For correlation analyses displayed in Figure 4, average GII values were calculated from 53BP1-stained CTCs in one blood sample. In Supplementary Figure S4, the highest GII value obtained from individual CTCs in one blood sample was chosen. Where GII values for baseline samples were missing, they were supplemented with GII data from HER2-stained CTCs.

### 4.4. Breast Cancer Cell Lines, Cultivation, and Treatment

All breast cancer cell lines except for HCC1937 were raised in DMEM (PAA Laboratories GmbH, Pasching, Austria). The following supplements were included: 10% fetal calf serum, modified Eagle medium containing non-essential amino acids (GIBCO/ Invitrogen GmbH, Karlsruhe, Germany), 5 mM L-glutamine (Biochrom, Berlin, Germany), 10 ng/ml epidermal growth factor (Sigma, St Louis, MO), and 4 mg/ml insulin (GIBCO). We used exactly the same stocks of the following cell lines as used and characterized in Concin et al [46] and Keimling et al [47]: MDA-MB-453 (MM453), T47D, BT20, MDA-MB-231 (MM231), Hs578t, MDA-MB-157 (MM157), and MDA-MB-436 (MM436) (all provided by University Clinic Ulm). The breast cancer cell lines MCF7 and MDA-MB-468 (MM468) were purchased from Cell Lines Services (CLS, Eppelheim Germany). Cell line ZR75-1 was derived from the same stock as the one used in Ireno et al [48] (provided by Experimental Pharmacology and Oncology, EPO, Berlin-Buch, Germany, after purchase from CLS). HCC1937 cells were obtained from the American Type Culture Collection (ATCC, CRL-2336™) and cultivated in RPMI (Gibco) with 15% fetal bovine serum. For experimental setups with drug treatments, the cells were seeded on day 0 to establish exponential growth overnight, followed by Eribulin treatment (1 nM) on day 1, medium changes with fresh Eribulin on days 3 and 6, and finally cell harvest on day 8 for Western blotting and cell fixation for immunofluorescence analysis. All cell cultures were regularly subjected to PCR-based test for Mycoplasma contamination.

### 4.5. Western Blot Analysis

Analysis of protein expression in breast cancer cell lines was performed exactly as described [48]. Briefly, proteins were extracted from freshly harvested cells; SDS polyacrylamide gel electrophoresis and Western transfer were performed with the extracts, followed by immunodetection using specific antibodies and chemiluminescence substrate. The following primary antibodies were used: anti-53BP1 (polyclonal rabbit, NB 100-304, Novus Biologicals) and anti-Vimentin (polyclonal rabbit, 10366-1-AP, Proteintech/Acris Antibodies GmbH/Origene, Herford, Germany). To control for loading, we reincubated the blots with mouse mAb anti-ß-actin (clone C4, sc-47778, Santa Cruz Biotechnology, Heidelberg, Germany). Peroxidase-conjugated secondary antibodies were purchased from Rockland Immunochemicals Inc., Pennsylvania, USA. To quantify protein band intensities, we used a ChemiDocMP system (Bio-Rad, München, Germany) and corrected the values for the proteins of interest with the values of the corresponding loading control.

### 4.6. Quantitative Immunofluorescence Microscopy

Breast cancer cell lines were grown on cover slips, fixed with 3.7% formaldehyde in PBS, permeabilized with 0.5% TritonX-100, and incubated with antibodies and nuclear DAPI-stain as described in Deniz et al [49]. Primary antibodies used for immunostaining were polyclonal rabbit antibodies against 53BP1 (NB100-304, Novus Biologicals) and mouse mAb anti-phospho-Histone H2A.X (Ser139, clone JBW301, Invitrogen, Darmstadt, Germany). As fluorescently labeled secondary antibodies, we used AF555 anti-rabbit and AF488 anti-mouse antibodies (Invitrogen). Immunofluorescence microscopy was performed using a BZ-9000 microscope (Keyence, Neu-Isenburg, Germany). Foci detection and quantification were carried out with BZ-II Analyzer software.

### 4.7. Statistical Analysis

Categorical data are described using absolute and relative frequencies, and between-group comparisons of categorical data were performed using the Χ^2^ test (or Fisher’s exact test in cases where the expected frequencies in any cell of 2 x 2 contingency tables were less than 5). The metric variables age (years) and body mass index (BMI, kg/m^2^) were described using median and range, and between-group comparisons were performed using the nonparametric Mann–Whitney U test. For comparisons regarding CTC enumerations and 53BP1 scorings in CTCs by CellSearch^®^ technology, Western blot quantification and nuclear foci scorings by immunofluorescence microscopy, we used Kruskal–Wallis-tests to test for overall differences among three or more independent groups, followed by pairwise comparisons with Mann–Whitney U tests in the case of statistical significance.

Progression-free survival (PFS) was analyzed using the Kaplan–Meier method, and survival data were illustrated using Kaplan–Meier survival plots. Between-group comparisons of PFS were performed with the log-rank test. All time-to-event intervals were measured from the date of recruitment to the DETECT IV B study to the date of the progress. If no progress was documented, the data were censored at the date of the last adequate follow-up assessment (clinical investigation and/or imaging).

All statistical analyses were performed using either GraphPad Prism software version 8.1.0 or SPSS version 24 (IBM Corp, Armonk, New York, USA). All statistical tests were two-sided; p values below 0.05 were considered significant, and there was no adjustment of significance levels for multiple testing. * *P* < 0.05, ** *P* < 0.01, *** *P* < 0.001.

## 5. Conclusions

Prognosis of MBC is poor, and targeted therapies are limited. However, significant numbers of CTCs are detectable in the blood of MBC patients that could be exploited as liquid biopsies. Eribulin is a promising drug against MBC with non-canonical microtubule-inhibitory and additional rather unexplored anti-cancerogenic mode-of-actions for which biomarkers are needed. Our study demonstrates that 53BP1, which promotes microtubule-dependent DNA mobility and repair, stains CTCs in patients with HR+ MBC. 53BP1 signals increased during Eribulin monotherapy and showed a trend to associate with progression-free survival. Altogether, the outcomes of this project may provide a new clue to MBC treatment response mechanisms and deliver a clinically feasible biomarker for early response prediction and/ therapeutic resistance.

## Figures and Tables

**Table 1:** Demographic and clinicopathological features of the analyzed MBC patients (N = 67) according to the HR status of the primary tumor (data available for 63 patients) and according to the HR status of metastases (data available for 43 patients).

Immunophenotype	Primary Tumor (N = 63)			Metastases (N = 43)		
	Triple-Negative *N (%)*	Luminal-Like (HR+) *N (%)*	*P*-Value ^2^	Triple-Negative *N (%)*	Luminal-Like (HR+) *N (%)*	*P*-Value ^2^
	15 (23.8%)	48 (76.2%)		14 (32.6%)	29 (67.4%)	
**Age (years)**	*Median (Range)*		0.272 ^3^	*Median (Range)*		0.613 ^3^
	65 (43–78)	62 (34–81)		64 (43–78)	63 (39–75)	
**BMI (kg/m^2^)**	*Median (Range)*		0.116 ^3^	*Median (Range)*		1.000 ^3^
	23.3 (18.0–39.7)	26.4 (18.4–40.6)		24.6 (18.6–39.7)	24.0 (19.4–35.3)	
**ECOG**	*N (%)*		0.004 ^4^	*N (%)*		0.035 ^4^
**0**	4 (26.7%)	35 (72.9%)		5 (35.7%)	22 (75.9%)	
**1**	9 (60.0%)	12 (25.0%)		7 (50.0%)	6 (20.7%)	
**2**	2 (13.3%)	1 (2.1%)		2 (14.3%)	1 (3.4%)	
**Histotype**	*N (%)*		0.549 ^4^	*N (%)*		0.537 ^4^
**Ductal**	11 (73.3%)	37 (77.1%)		9 (64.3%)	23 (79.3%)	
**Lobular**	1 (6.7%)	6 (12.5%)		2 (14.3%)	3 (10.3%)	
**Mixed and Other**	3 (20.0%)	5 (10.4%)		3 (21.4%)	3 (10.3%)	
**Grading**	*N (%)*		0.004 ^4^	*N (%)*		0.041 ^4^
**1**	0 (0.0%)	1 (2.1%)		0 (0.0%)	0 (0.0%)	
**2**	4 (26.7%)	31 (64.6%)		4 (28.6%)	17 (58.6%)	
**3**	11 (73.3%)	11 (22.9%)		9 (64.3%)	9 (31.0%)	
**Unknown**	0 (0.0%)	5 (10.4%)		1 (7.1%)	3 (10.3%)	
**Metastatic site—locally advanced ^1^**	*N (%)*		0.028 ^5^	*N (%)*		0.252 ^5^
**Yes**	6 (40.0%)	6 (12.5%)		5 (35.7%)	5 (17.2%)	
**No**	9 (60.0%)	42 (87.5%)		9 (64.3%)	24 (82.8%)	
**Metastatic site—bone ^1^**	*N (%)*		0.012 ^4^	*N (%)*		0.507 ^5^
**Yes**	6 (40.0%)	36 (75.0%)		8 (57.1%)	20 (69.0%)	
**No**	9 (60.0%)	12 (25.0%)		6 (42.9%)	9 (31.0%)	
**Metastatic site—visceral ^1^**	*N (%)*		0.291 ^5^	*N (%)*		0.055 ^5^
**Yes**	10 (66.7%)	39 (81.3%)		8 (57.1%)	25 (86.2%)	
**No**	5 (33.3%)	9 (18.8%)		6 (42.9%)	4 (13.8%)	
**Metastatic site—CNS ^1^**	*N (%)*		0.039 ^5^	*N (%)*		0.094 ^5^
**Yes**	3 (20.0%)	1 (2.1%)		3 (21.4%)	1 (3.4%)	
**No**	12 (80.0%)	47 (97.9%)		11 (78.6%)	28 (96.6%)	
**Line of chemotherapeutical treatment (in metastatic setting)**	*N (%)*		0.741 ^4^	*N (%)*		0.899 ^4^
**1**	6 (40.0%)	27 (56.3%)		8 (57.1%)	15 (51.7%)	
**2**	5 (33.3%)	13 (27.1%)		4 (28.6%)	8 (27.6%)	
**3 or more**	3 (20.0%)	8 (16.7%)		2 (14.3%)	6 (20.7%)	
**Unknown**	1 (6.7%)	0 (0.0%)		0 (0.0%)	0 (0.0%)	

Abbreviations: BMI, body mass index; ECOG, Eastern Cooperative Oncology Group performance status; CNS, central nervous system. ^1^ Note that some patients carried metastases at multiple sites. Among the primary tumor patients for which the HR status was determined both in the primary tumor as well as in the metastasic site (TNBC, N=11; HR+: N=28), 81.8% of patients with triple-negative primary tumor had triple-negative metastases, and 82.1% of patients with HR+ primary tumor had HR+ metastases. ^2^ Without ‘unknowns’. ^3^ Mann–Whitney U test. ^4^ Chi-square test. ^5^ Fisher’s exact test.

## Supplementary Materials

The following are available online at https://www.mdpi.com/2072-6694/12/4/930/s1, Figure S1: CTC numbers and CTC-specific 53BP1 positivity. Figure S2. Recruitment of patients with HR+ and HR- primary tumors or metastases and individual patient scorings. Figure S3. Focal accumulation of 53BP1 and γH2AX in the nucleus of cell lines. Figure S4. Comparison of maximum genomic integrity in individual CTCs versus 53BP1 scores. Figure S5. Uncropped Western Blot Figures.
