# Supplementary material for: 53BP1 Accumulation in Circulating Tumor Cells Identifies Chemotherapy-Responsive Metastatic Breast Cancer Patients
Source: Cancers (Basel). 2020 Apr 9;12(4):930. doi: 10.3390/cancers12040930 (PMC7226269; doi:10.3390/cancers12040930)
Supplement: Supplementary file 1 [file cancers-12-00930-s001.pdf]

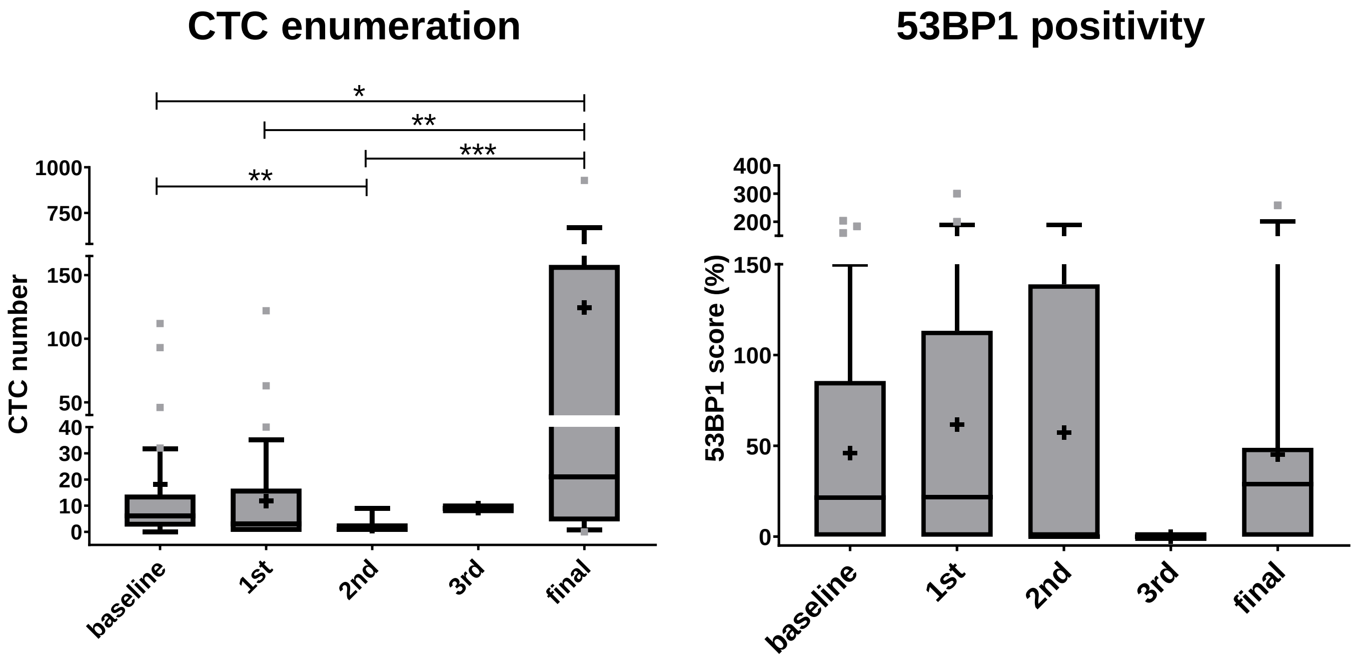

Figure S1.

CTC numbers and CTC-specific 53BP1 positivity. CTCs from the MBC patients recruited in the Eribulin arm of the DETECT IV trial were enumerated and 53BP1 staining evaluated. Data are shown in box plots with mean value (dot), median (line) and 95% Confidence Intervals (CI) (whiskers). For numbers of independent blood samples (N) obtained during different visits see Fig. 1. *P<0.05, **P<0.01, ***P<0.001, Kruskal-Wallis test, Mann-Whitney test.

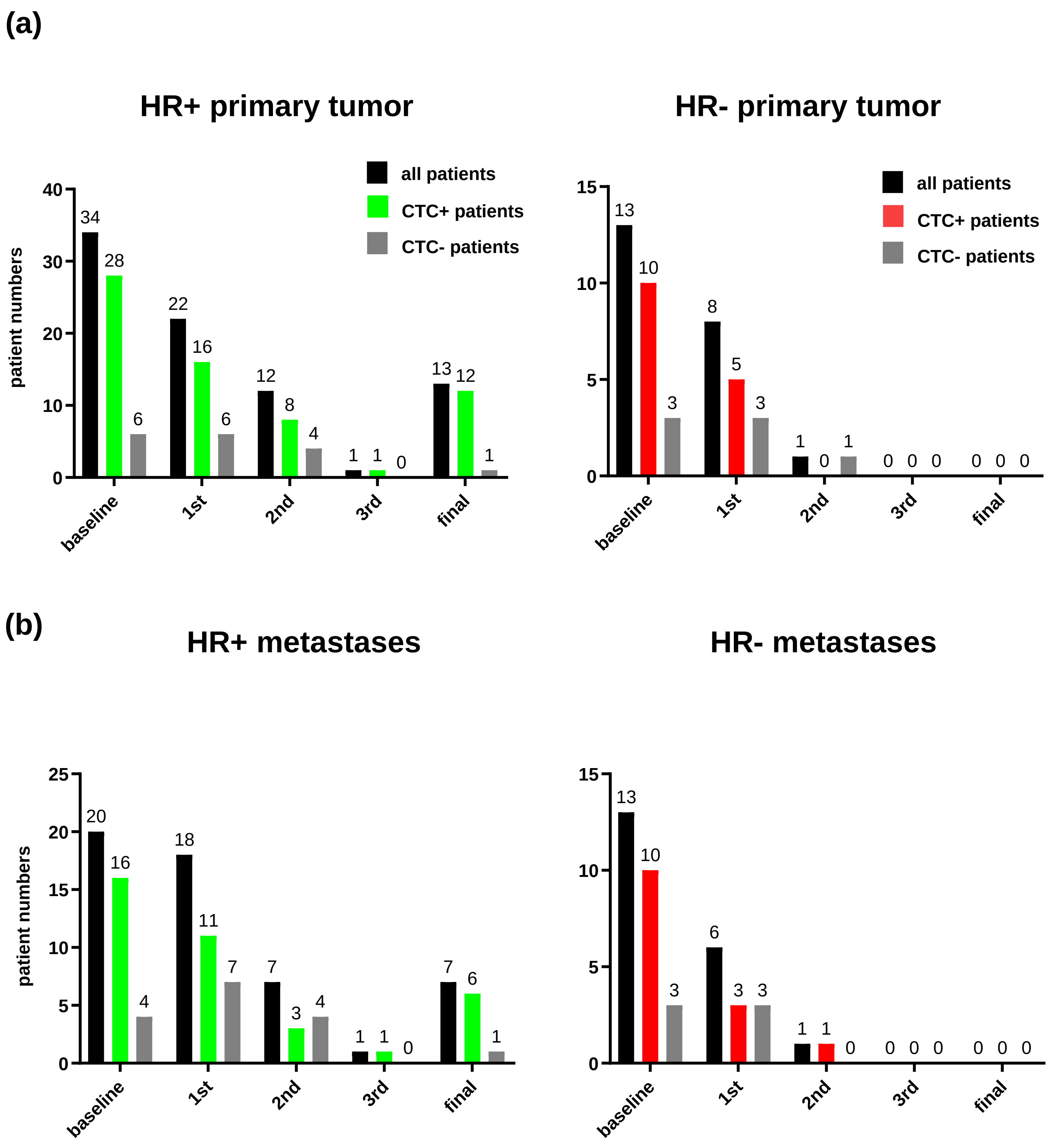
(a)
HR+ primary tumor
all patients
CTC+ patients
CTC- patients
patient numbers
40
30
20
10
0
34
28
6
22
16
6
12
8
4
1
1
0
13
12
1
baseline
1st
2nd
3rd
final
HR- primary tumor
all patients
CTC+ patients
CTC- patients
15
10
5
0
13
10
3
8
5
3
1
0
1
0 0 0
0 0 0
baseline
1st
2nd
3rd
final
(b)
HR+ metastases
patient numbers
25
20
15
10
5
0
20
16
4
18
11
7
7
3
4
1
1
0
7
6
1
baseline
1st
2nd
3rd
final
HR- metastases
15
10
5
0
13
10
3
6
3
3
1
1
0
0 0 0
0 0 0
baseline
1st
2nd
3rd
final

(c)

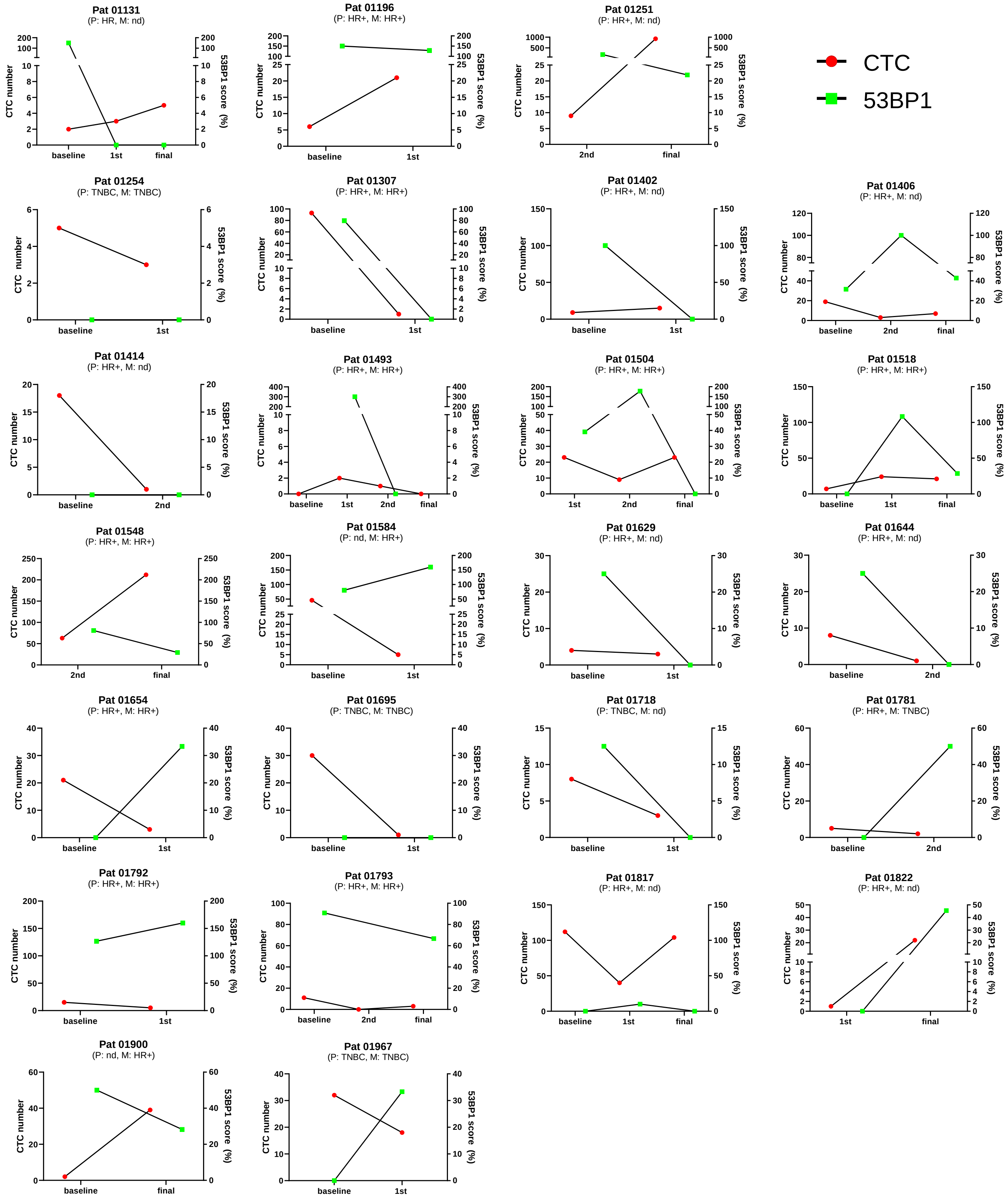

Figure S2.

Recruitment of patients with HR+ and HR- primary tumors or metastases and individual patient scorings. The numbers of MBC patients recruited at the different visits are indicated by black columns, the numbers of CTC+ and CTC- patients among them by red and grey columns, respectively.

(a) Numbers of patients with HR+ versus HR- primary tumors (ad Figure 2a).

(b) Numbers of patients with HR+ versus HR- metastases (ad Figure 2b).

(c) CTC numbers and 53BP1 scores for each patient, for whom 53BP1 scores were determined at least for two visits; P, primary tumor; M, metastasis, nd, not determined.

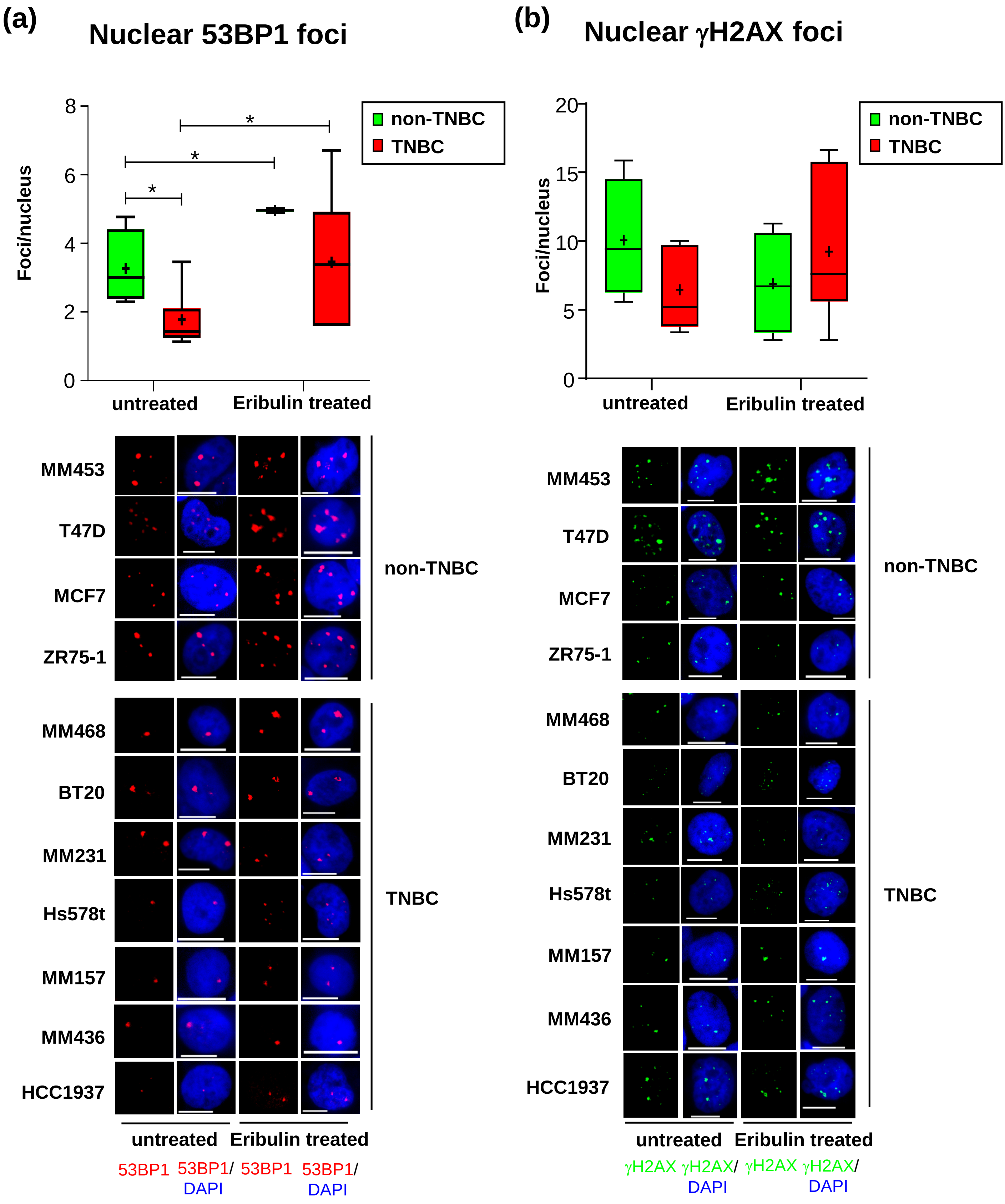

Figure S3.
Focal accumulation of 53BP1 and γH2AX in the nucleus of cell lines.
(a) 53BP1 foci counts for mock and Eribulin treatment are graphically shown. Box plots in the upper panel show mean values (cross), median (line) and 95% CI (whiskers) for untreated and Eribulin-treated non-TNBC (N=4) and TNBC cell lines (N=7). Altogether, ~300-900 nuclei per sample were scored in two independent experiments each. *P<0.05, Kruskal-Wallis test, Mann-Whitney test. Representative immunofluorescence images are shown in the lower panel. Scale bar, 10 μm.
(b) γH2AX foci were scored by quantitative immunofluorescence microscopy from ~100-600 nuclei per sample in two experiments, graphically displayed and statistically evaluated as in (a). Exemplary images of DAPI-stained nuclei (blue) with γH2AX foci (green) are displayed below.

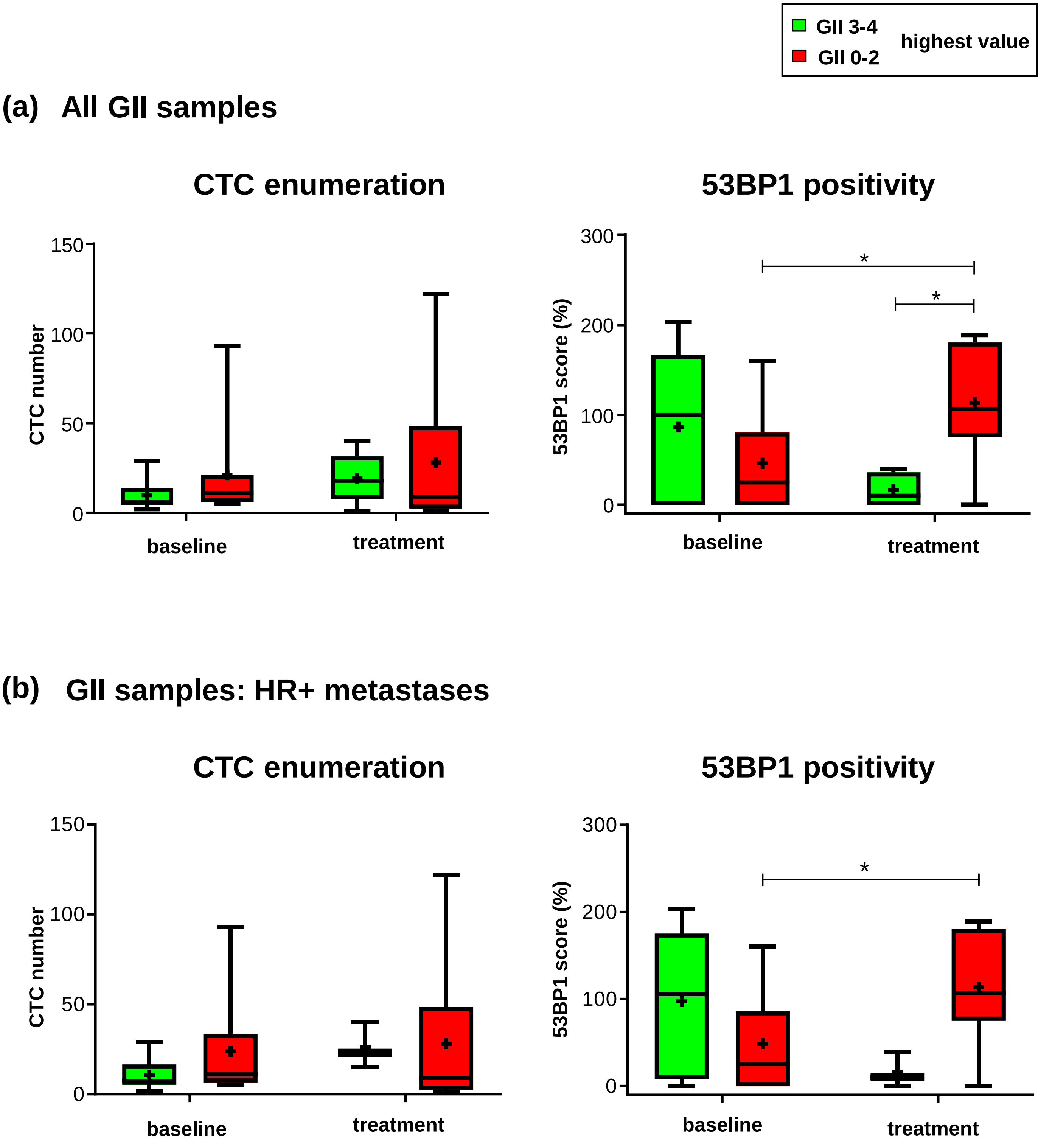

Figure S4.

Comparison of maximum genomic integrity in individual CTCs versus 53BP1 scores.

GII values were determined for single CTCs as in Fig. 4 but the highest values scored rather than average values per sample (see Fig. 4) compared with sample-specific 53BP1 scores. The left graphs display CTC numbers with high versus low genomic integrity for blood samples collected during baseline and treatment visits 1-2. The right panels show 53BP1 scores for these four groups each. Box plots show mean values (cross), median (line) and 95% CI (whiskers). *$P<0.05$, Mann-Whitney test.

(a) All GII samples.

Baseline: GII 3-4, N=9 and GII 0-2, N=11; treatment visits 1-2: GII 3-4, N=5 and GII 0-2, N=6.

(b) GII samples for HR+ metastases.

Baseline: GII 3-4, N=8 and GII 0-2, N=9; treatment visits 1-2: GII 3-4, N=3 and GII 0-2, N=6.

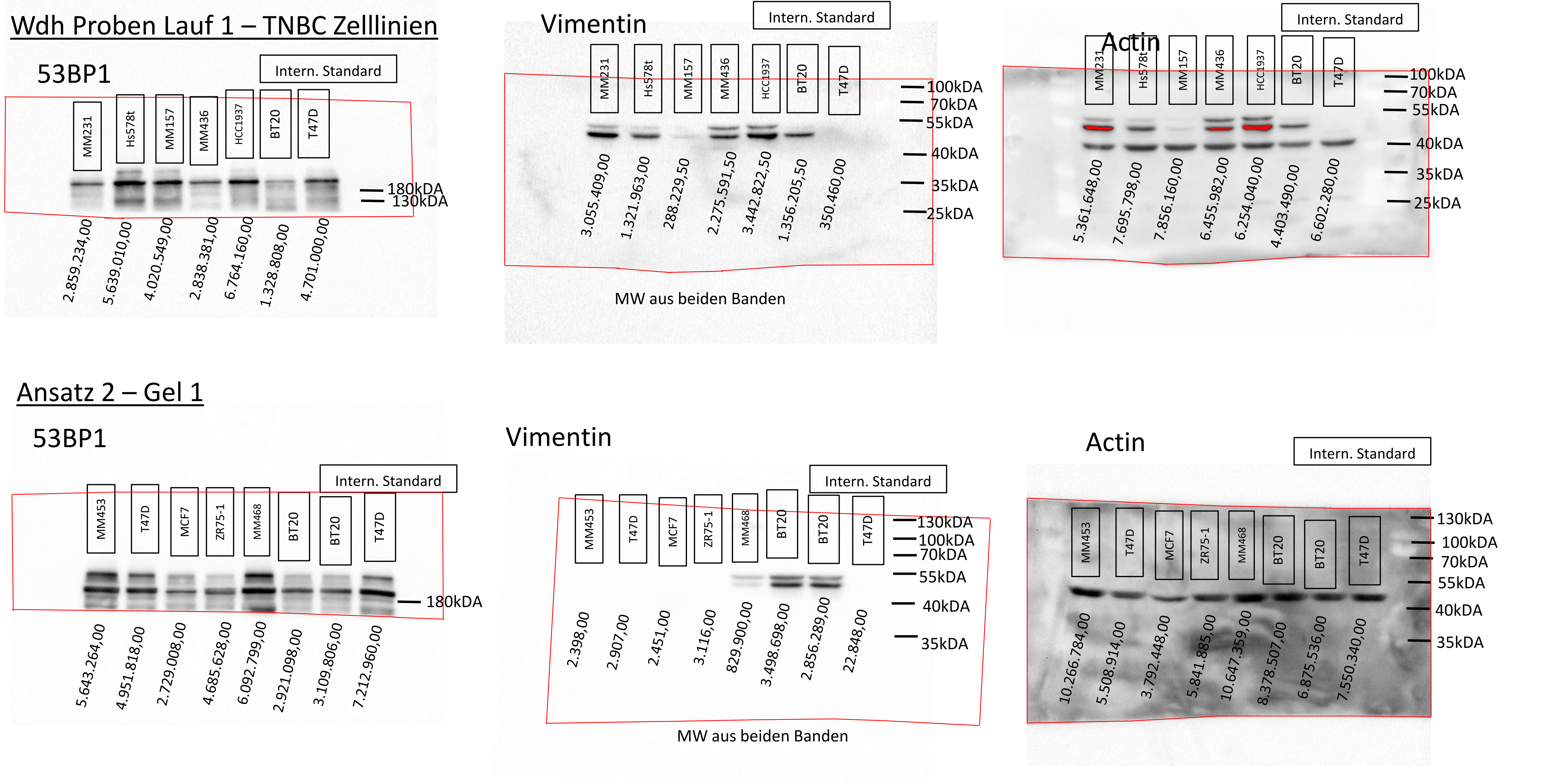

Figure S5. Uncropped Western Blot Figures.
